# Rescue of myocardial energetic dysfunction in diabetes through the correction of mitochondrial hyperacetylation by honokiol

**DOI:** 10.1172/jci.insight.140326

**Published:** 2020-09-03

**Authors:** Matthew Kerr, Jack J. Miller, Dharendra Thapa, Sophie Stiewe, Kerstin N. Timm, Claudia N. Montes Aparicio, Iain Scott, Damian J. Tyler, Lisa C. Heather

**Affiliations:** 1 Department of Physiology, Anatomy and Genetics, University of Oxford, Oxford, United Kingdom.; 2 Oxford Centre for Clinical Magnetic Resonance Research, John Radcliffe Hospital, Oxford, United Kingdom.; 3 Department of Physics, University of Oxford, Oxford, United Kingdom.; 4 Vascular Medicine Institute, University of Pittsburgh, Pittsburgh, Pennsylvania, USA.

**Keywords:** Cardiology, Metabolism, Mitochondria

## Abstract

Cardiac energetic dysfunction has been reported in patients with type 2 diabetes (T2D) and is an independent predictor of mortality. Identification of the mechanisms driving mitochondrial dysfunction, and therapeutic strategies to rescue these modifications, will improve myocardial energetics in T2D. We demonstrate using ^31^P-magnetic resonance spectroscopy (^31^P-MRS) that decreased cardiac ATP and phosphocreatine (PCr) concentrations occurred before contractile dysfunction or a reduction in PCr/ATP ratio in T2D. Real-time mitochondrial ATP synthesis rates and state 3 respiration rates were similarly depressed in T2D, implicating dysfunctional mitochondrial energy production. Driving this energetic dysfunction in T2D was an increase in mitochondrial protein acetylation, and increased ex vivo acetylation was shown to proportionally decrease mitochondrial respiration rates. Treating T2D rats in vivo with the mitochondrial deacetylase SIRT3 activator honokiol reversed the hyperacetylation of mitochondrial proteins and restored mitochondrial respiration rates to control levels. Using ^13^C-hyperpolarized MRS, respiration with different substrates, and enzyme assays, we localized this improvement to increased glutamate dehydrogenase activity. Finally, honokiol treatment increased ATP and PCr concentrations and increased total ATP synthesis flux in the T2D heart. In conclusion, hyperacetylation drives energetic dysfunction in T2D, and reversing acetylation with the SIRT3 activator honokiol rescued myocardial and mitochondrial energetics in T2D.

## Abstract 2

Pharmacologically targeting mitochondrial acetylation provides a mechanism to rescue impaired myocardial energy generation in type 2 diabetes.

## Graphical abstract

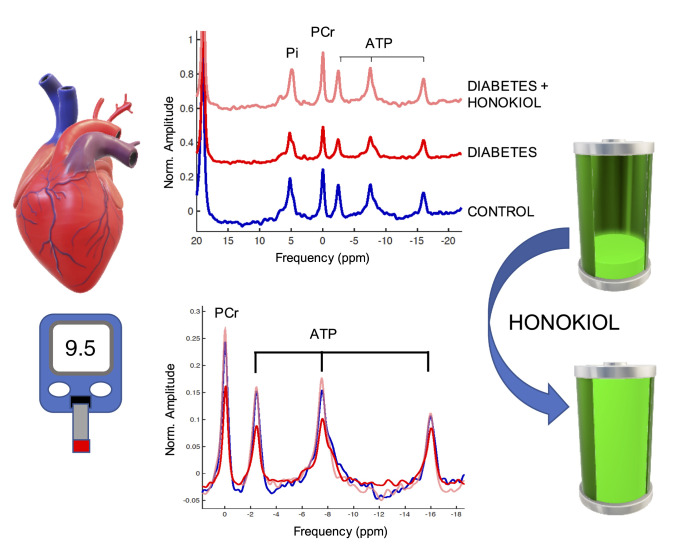

## Introduction

The heart requires more energy per gram of tissue than any other organ. Decreased phosphocreatine (PCr)/ATP ratios have been reported in patients with type 2 diabetes (T2D) (1), and, in patients with heart failure, this has been shown to be an independent predictor of mortality (2). Therefore, therapeutic strategies that improve myocardial energetics may provide a route to reduce cardiovascular mortality in diabetes. In silico modeling indicates that decreased cardiac energetics can be traced to decreased mitochondrial ATP synthesis (3). However, few studies have simultaneously investigated myocardial and mitochondrial energetics. Isolating the mechanisms that drive myocardial energetic dysfunction, particularly at the early stages of T2D, and identifying compounds that reverse this dysfunction are of great interest.

A number of mechanisms have been proposed to explain mitochondrial dysfunction in diabetes, which include increased reactive oxygen species leading to oxidative damage, increased uncoupling, abnormal calcium handling, and altered substrate utilization (4). Of particular interest are recent studies linking polymorphisms in the mitochondrial protein deacetylase, SIRT3, with metabolic syndrome (5). Protein acetylation refers to the addition of an acetyl moiety from acetyl-CoA to the ε-amino group of a lysine residue, with an estimated 35% of mitochondrial proteins acetylated on one or more lysine residues (6). SIRT3 is the mitochondrially localized member of the NAD^+^-dependent sirtuin family and regulates the deacetylation of mitochondrial proteins (7). Mitochondrial protein acetylation is generally associated with a reduction in enzymatic activity, as demonstrated for enzymes involved in the electron transport chain and the Krebs cycle (7–10). Excessive fatty acid (FA) oxidation, such as that which occurs in T2D, generates an intramitochondrial environment with a relative abundance of acetyl-CoA and a relative scarcity of NAD^+^ (11), conducive to hyperacetylation. Hyperacetylation of cardiac mitochondrial proteins has been observed after high-fat diet feeding (12) and in type 1 diabetes (13), as well as within the hearts of obese patients (14); however, the physiological consequences for myocardial and mitochondrial energetics remains unclear.

Pharmacological approaches to decrease mitochondrial acetylation have received attention and have focused on enhancing SIRT3 activity. Strategies to elevate intramitochondrial NAD^+^ have improved mitochondrial function (15, 16) but can have off-target effects on nuclear sirtuins and cellular redox balance, whereas small molecule activators, such as resveratrol, have been shown to possess only modest SIRT specificity (17). However, in recent years a small biphenolic molecule, honokiol, which demonstrates good bioavailability and specificity for SIRT3 activation (18), has been identified. Honokiol has been demonstrated in vivo to correct acetylation of mitochondrial superoxide dismutase in models of cardiac hypertrophy and doxorubicin-induced cardiomyopathy, reducing fibrosis and wall thickness (18, 19). Whether it can also correct hyperacetylation of metabolic enzymes to correct mitochondrial dysfunction and rescue energetic impairment in T2D has not been investigated.

Using a combination of ^31^P-magnetic resonance spectroscopy (^31^P-MRS) on the perfused heart and isolated mitochondria, mitochondrial respiration, and in vivo hyperpolarized ^13^C-MRS, we set out to investigate the relationship between myocardial energetics and mitochondrial energetics and identify key mechanisms that could be targeted therapeutically to improve energetics. We propose that myocardial energetics are impaired early in the progression of T2D, and we have localized this to decreased mitochondrial respiration and ATP synthesis rates. We demonstrate that energetic dysfunction arises from excessive mitochondrial protein acetylation, which we can pharmacologically reverse using honokiol. This work demonstrates that honokiol, a mitochondrial deacetylase activator, restores mitochondrial function, and rescues myocardial energetic dysfunction in T2D.

## Results

### T2D rodent hearts were energetically impaired despite normal PCr/ATP ratios.

**Figure 1:**
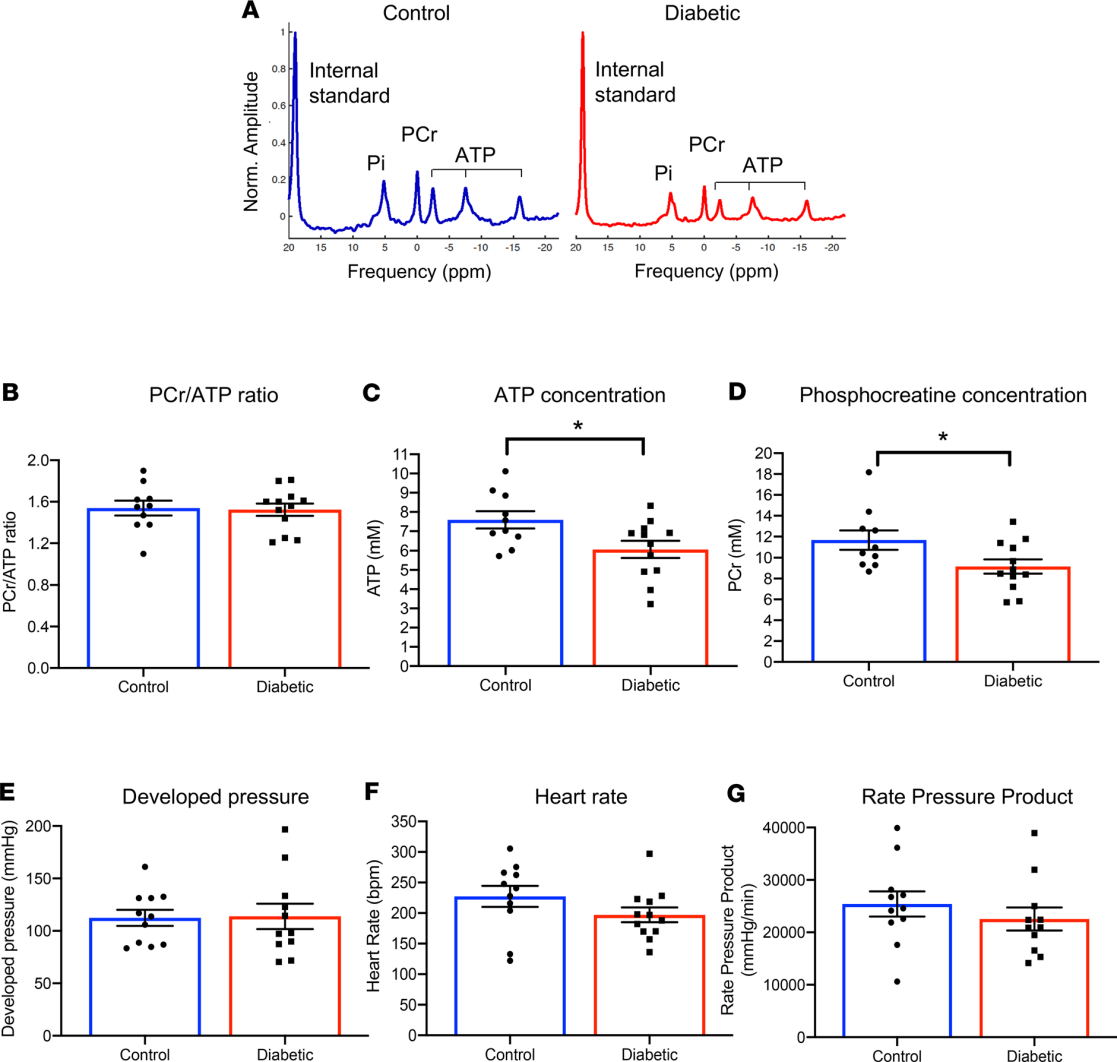
T2D hearts are energetically impaired despite normal PCr/ATP ratios. Myocardial energetics were measured using ^31^P-spectroscopy in the intact contracting heart, with representative spectra shown (**A**). PCr/ATP ratios, absolute ATP, and absolute PCr concentrations from control and T2D hearts (**B–D**). Cardiac function, as assessed by developed pressure, heart rate, and rate pressure product, in control and T2D hearts (**E–G**). *P < 0.05 vs. control, analyzed using a 2-tailed parametric unpaired *t* test. T2D, type 2 diabetes; PCr, phosphocreatine.

T2D rats exhibited mild hyperglycemia (29% increase in fasting blood glucose), increased adiposity, and increased body weight (Supplemental Table 1; supplemental material available online with this article; https://doi.org/10.1172/jci.insight.140326DS1). To investigate myocardial energetics in T2D, high-energy phosphates were measured in actively contracting hearts using ^31^P-MRS. We found no significant difference in PCr/ATP ratios in T2D hearts, compared with control hearts (Figure 1, A and B), the primary clinical measurement of myocardial energetics that can be made in patients (1, 2, 20). The advantage of assessment in the perfused heart is that we can use an internal standard within the ventricle to allow the absolute quantification of ATP and PCr concentrations. T2D hearts had significant decreases in the concentrations of both ATP and PCr, by 20% and 22%, respectively (Figure 1, C and D), which were masked when only the PCr/ATP ratio was evaluated. These changes in energetics occurred independently of changes in cardiac function, with no significant differences in developed pressure, heart rate, or rate pressure product in this early-stage model of T2D (Figure 1, E–G).

### T2D cardiac mitochondria have reduced respiration and ATP synthesis rates.

**Figure 2:**
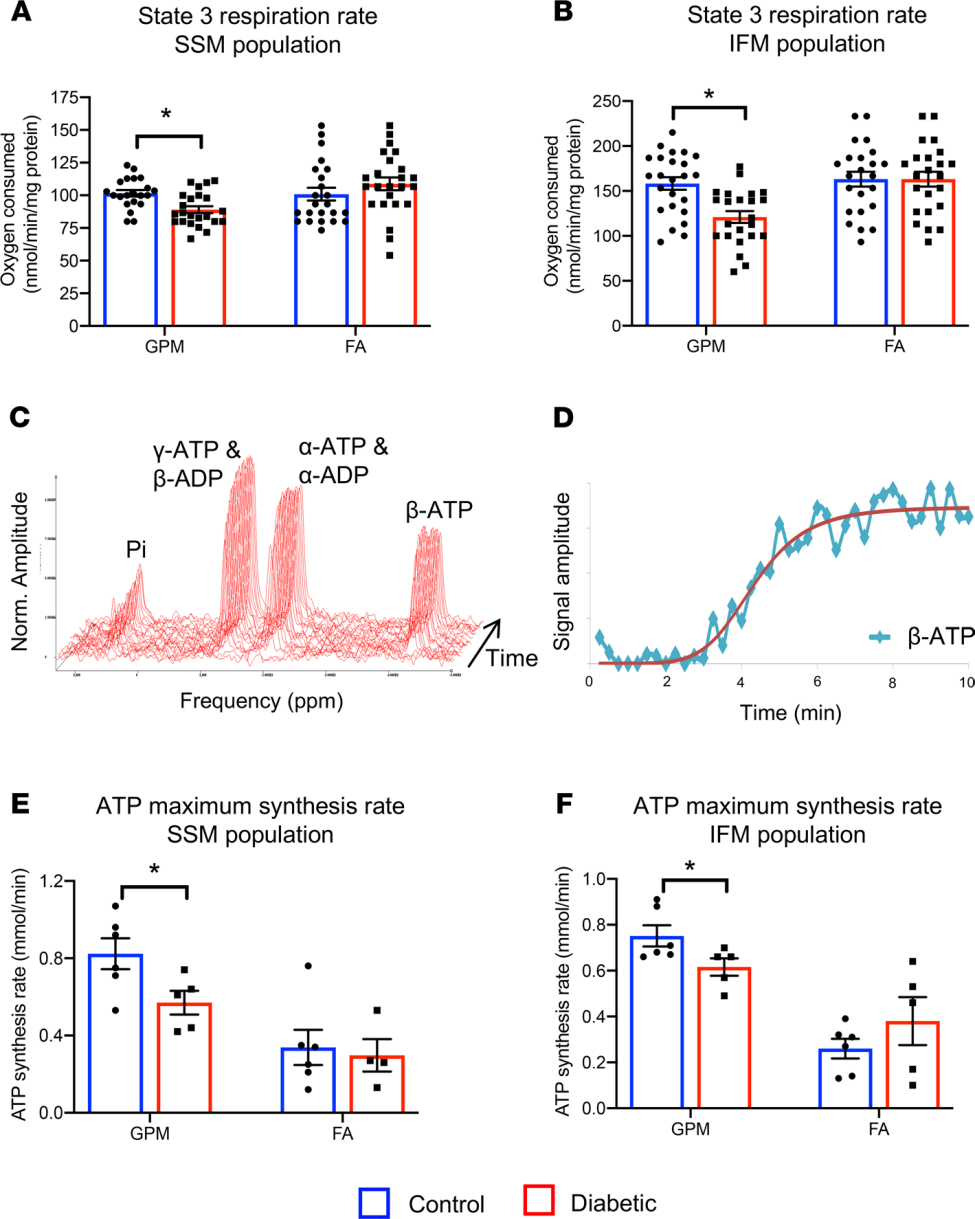
T2D mitochondria have impaired respiration and real-time ATP synthesis rates. State 3 respiration rates in control and T2D SSM and IFM mitochondrial populations, metabolizing carbohydrates and amino acid (GPM) or FA (**A** and **B**). Mitochondrial ATP synthesis rates were measured in respiring mitochondria using ^31^P-spectroscopy (representative spectra shown and fitted for quantification in **C** and **D**). Mitochondrial maximum ATP synthesis rates in control and T2D SSM and IFM populations, metabolizing carbohydrates and amino acid (GPM) or FAs (**E** and **F**). *P < 0.05 vs. control, analyzed using a 2-way ANOVA with Holm-Sidak’s post hoc correction. T2D, type 2 diabetes; SSM, subsarcolemmal; IFM, interfibrillar; GPM, glutamate, pyruvate, malate; FAs, fatty acids.

Mitochondria generate over 95% of the ATP for the heart, making them the logical source of impaired energetics. Mitochondrial state 3 respiration revealed that T2D mitochondria had a 12% decrease in ADP-stimulated respiration rates in the subsarcolemmal (SSM) population and a 24% decrease in the interfibrillar (IFM) population, when respiring on a carbohydrate and amino acid mix (glutamate, pyruvate, and malate [GPM]) (Figure 2, A and B). T2D mitochondria displayed no reduction in state 3 respiration rates when respiring with the addition of FAs.

To determine whether reduced rates of mitochondrial oxygen consumption resulted in reduced rates of ATP production, ^31^P-MRS experiments were conducted on isolated mitochondria to measure rates of mitochondrial ATP synthesis in real time (Figure 2, C and D). Maximal ATP synthesis rates when respiring on GPM were decreased by 31% in SSM and 18% in IFM in T2D hearts, compared with controls (Figure 2, E and F). This decrease in the maximal rate of synthesis of ATP occurred independently of a change in total ATP synthesized (Supplemental Figure 1). In agreement with the respiration data, when respiring with FA as cosubstrate there were no significant differences in maximal ATP synthesis rates (Figure 2, E and F) or total ATP synthesized (Supplemental Figure 1) in the mitochondria isolated from T2D hearts.

### Mitochondrial acetylation correlates with reduced respiratory rates.

**Figure 3:**
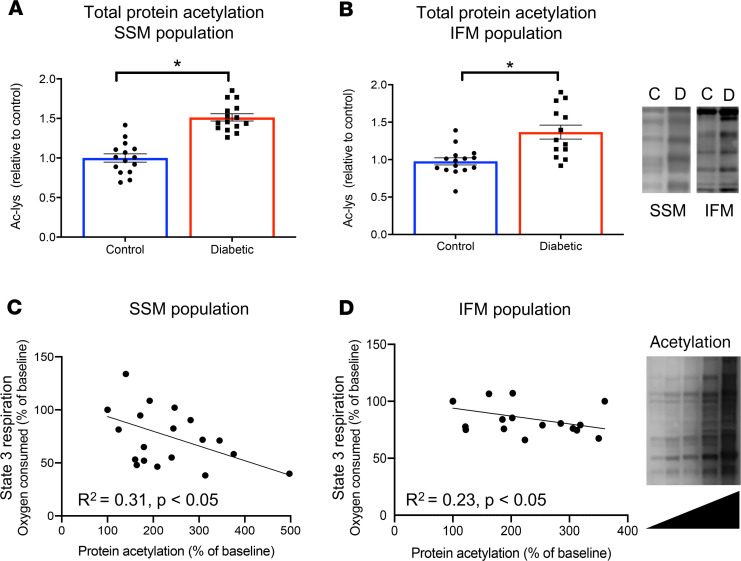
T2D mitochondria are hyperacetylated, with a negative correlation between acetylation and respiratory rate. Total protein acetylation (Ac-lys) in the SSM and IFM mitochondrial populations from control (C) and T2D (D) hearts (**A** and **B**). Increased in vitro acetylation of control mitochondria was associated with decreased state 3 respiration rates, when metabolizing carbohydrates and amino acid (**C** and **D**), assessed using simple linear regression. *P < 0.05 vs. control, analyzed using a 2-tailed parametric unpaired *t* test. T2D, type 2 diabetes; SSM, subsarcolemmal; IFM, interfibrillar.

Mitochondrial enzymes have been shown to be regulated posttranslationally via acetylation, with increased levels of mitochondrial protein acetylation reported in obesity (12, 14). We questioned whether hyperacetylation may be involved in the reduced respiratory rates observed in T2D mitochondria. Total mitochondrial protein acetylation was 50% higher in both populations of T2D mitochondria compared with control mitochondria (Figure 3, A and B). To determine if this hyperacetylation decreases respiration, the effect of in vitro hyperacetylation was determined in control mitochondria. Hyperacetylation was found to negatively correlate with state 3 respiratory capacity in both SSM and IFM populations when metabolizing GPM (Figure 3, C and D). Using this linear relationship, we could calculate that hyperacetylation of control mitochondria by 50%, to mimic the degree of hyperacetylation we found in T2D mitochondria, decreased respiratory capacity by between 10% and 14%. This depression of respiration with in vitro hyperacetylation was of a similar order of magnitude to that observed in native T2D mitochondria.

### Pharmacological deacetylation recovers dysfunctional mitochondrial respiration in T2D.

**Figure 4:**
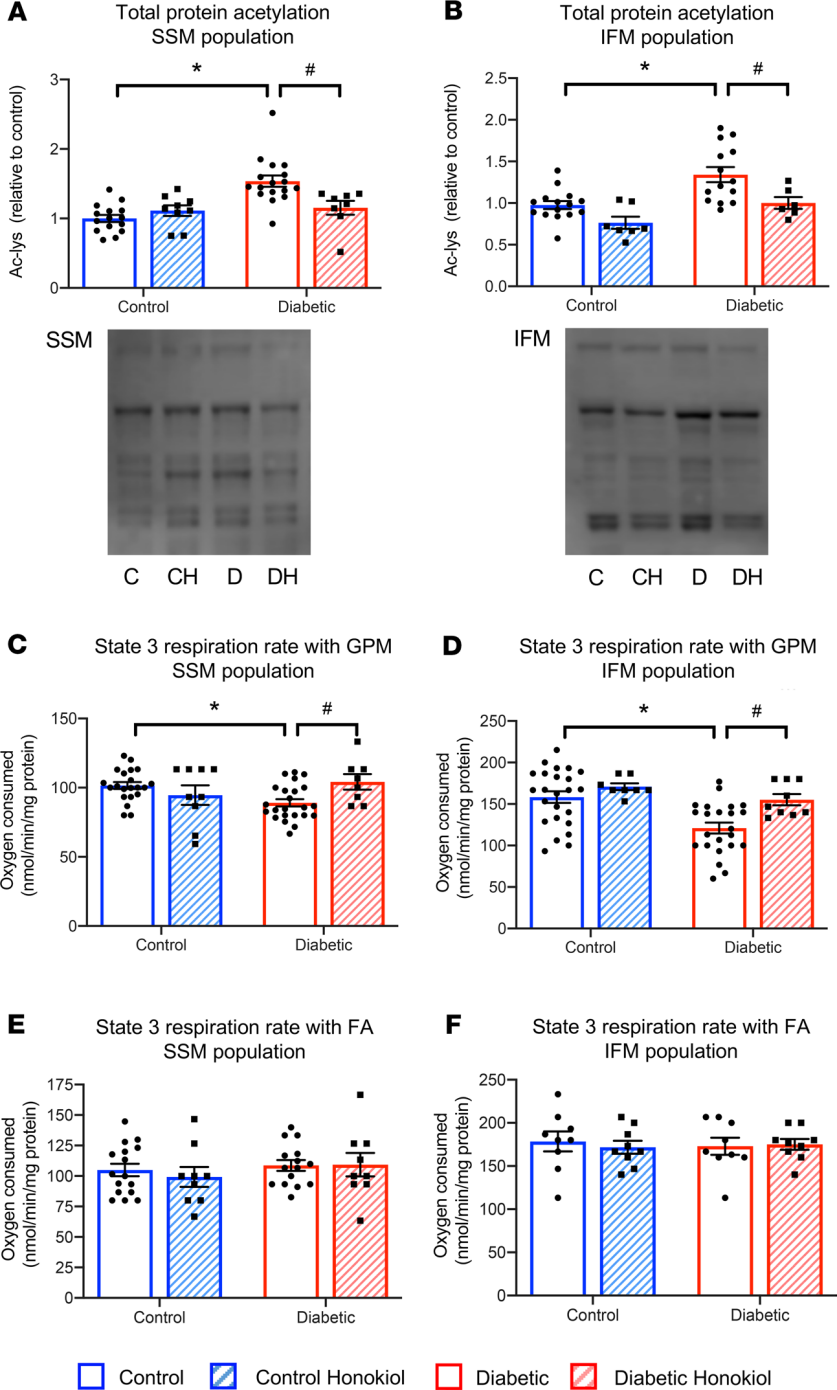
Honokiol recovers mitochondrial function in T2D. Total protein acetylation (Ac-lys) in the SSM and IFM mitochondrial populations from control and T2D hearts, with and without honokiol treatment (**A** and **B**). Example acetylation blots shown below graphs for control (C), control honokiol (CH), diabetic (D), and diabetic honokiol (DH) groups. State 3 respiration rates in control and T2D mitochondria, with and without honokiol treatment, metabolizing carbohydrates and amino acid (GPM, **C** and **D**) or FA substrates (**E** and **F**). *P < 0.05 vs. control, ^#^*P* < 0.05 vs. diabetic untreated, analyzed using a 2-way ANOVA with Holm-Sidak’s post hoc correction. T2D, type 2 diabetes; SSM, subsarcolemmal; IFM, interfibrillar; GPM, glutamate, pyruvate, malate; FAs, fatty acids.

Based on this relationship between hyperacetylation and reduced state 3 respiratory capacity, we investigated whether pharmacological correction of protein acetylation in T2D could rescue mitochondrial dysfunction. To investigate this, honokiol, an activator of the mitochondrial deacetylase SIRT3, was administered to T2D and control rats for 10 days. In vivo honokiol treatment decreased mitochondrial acetylation in T2D hearts by 25% in both SSM and IFM (Figure 4, A and B), correcting the hyperacetylation back to control levels. The improvement in acetylation resulted in improved mitochondrial function in honokiol-treated T2D hearts. State 3 respiration rates with GPM were significantly increased in honokiol-treated T2D hearts, increasing by 17% and 28% in SSM and IFM, respectively, back to control levels (Figure 4, C and D). In agreement with the normal FA state 3 respiration in T2D mitochondria, honokiol had no effect when FAs were included in the respiration media (Figure 4, E and F). This improvement in mitochondrial acetylation occurred independently of any changes in blood glucose, heart weight, body weight, adiposity (Supplemental Table 1), or SIRT3 protein levels (Supplemental Figure 2A).

**Figure 5:**
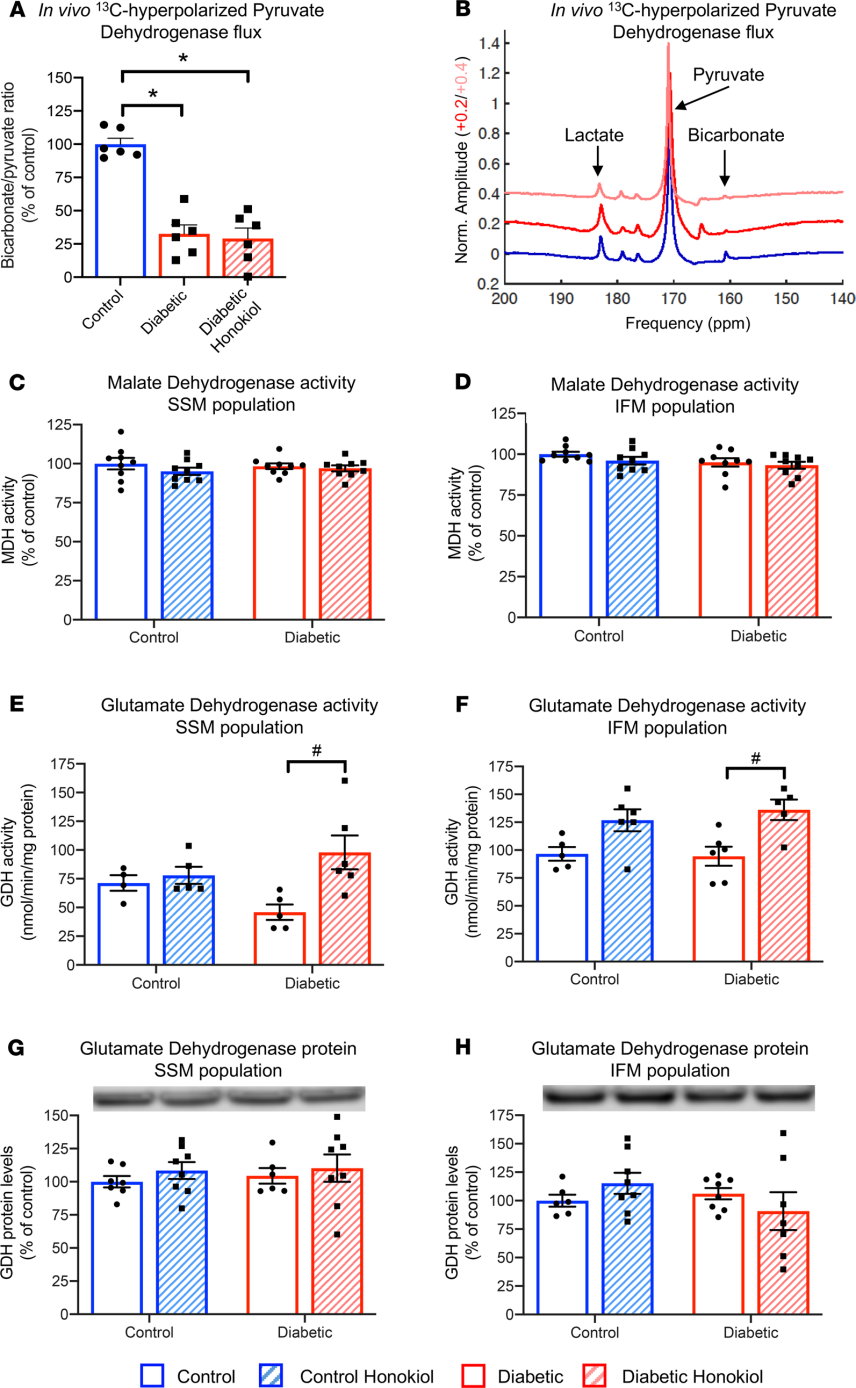
Honokiol improves flux through GDH. In vivo pyruvate dehydrogenase flux measured using ^13^C-hyperpolarized MR spectroscopy in control, T2D, and honokiol-treated T2D rats, with example spectra for control (blue), diabetic (red), and diabetic rats treated with honokiol (pink) (**A** and **B**). Malate dehydrogenase (**C** and **D**) and GDH (**E** and **F**) activities in SSM and IFM mitochondrial populations from control and T2D hearts, with and without honokiol treatment. GDH protein levels in SSM and IFM mitochondrial populations from control and T2D hearts, with and without honokiol treatment (**G** and **H**). *P < 0.05 vs. control, ^#^*P* < 0.05 vs. diabetic untreated. (**A**) One-way ANOVA with Tukey’s post hoc correction; all other panels analyzed using a 2-way ANOVA with Holm-Sidak’s post hoc correction. GDH, glutamate dehydrogenase, T2D, type 2 diabetes; SSM, subsarcolemmal; IFM, interfibrillar.

Honokiol improved respiration when respiring on excess GPM but had no effect when FAs were included, which indicated that honokiol was targeting an enzyme exclusive to GPM respiration. This localized the regulation to the entry points of GPM into the Krebs cycle, which are all known targets of SIRT3, namely, pyruvate dehydrogenase (PDH), malate dehydrogenase (MDH), and glutamate dehydrogenase (GDH). PDH activity was assessed both in vivo using hyperpolarized ^13^C-MRS and at the level of the isolated mitochondria. T2D decreased PDH activity both in vivo and ex vivo; however, honokiol had no effect on PDH flux (Figure 5, A and B, and Supplemental Figure 2B). Similarly, MDH activity was not modified by honokiol treatment (Figure 5, C and D). In contrast, GDH activity was increased by honokiol in T2D mitochondria in both SSM (2-fold) and IFM (44%) populations (Figure 5, E and F). This increase in GDH activity occurred independently of changes in GDH protein levels (Figure 5, G and H).

### Honokiol deacetylates GDH.

**Figure 6:**
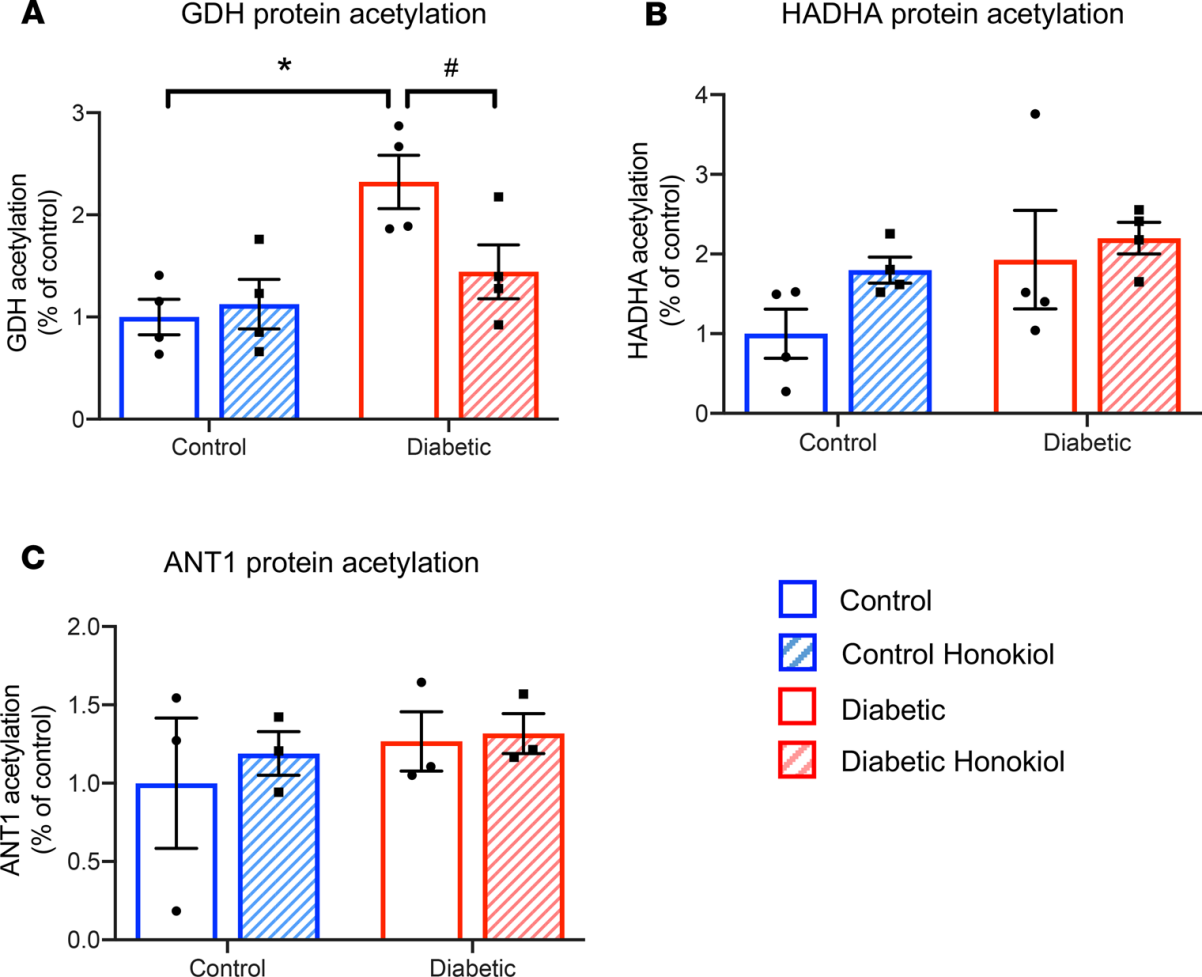
Honokiol selectively deacetylates GDH in T2D. Acetylation of GDH (**A**), hydroxyacyl-CoA dehydrogenase trifunctional multienzyme complex subunit α (HADHA) (**B**), and ADP/ATP translocase 1 (ANT1) (**C**) in SSM mitochondria. *P < 0.05 vs. control, ^#^*P* < 0.05 vs. diabetic untreated, analyzed using a 2-way ANOVA with Holm-Sidak’s post hoc correction. See Supplemental Figure 3 for example blots. GDH, glutamate dehydrogenase, T2D, type 2 diabetes; SSM, subsarcolemmal.

We set out to investigate if increased GDH activity was due to deacetylation of this enzyme. To confirm we could identify increased mitochondrial acetylated peptides in our T2D model, we looked at known targets from the literature. In agreement with others findings (21), we found GDH hyperacetylated in T2D and hydroxyacyl-CoA dehydrogenase trifunctional multienzyme complex subunit α (HADHA) trended toward being hyperacetylated (*P* = 0.09) by T2D (Figure 6, A and B). Changes in acetylation were not uniform across all mitochondrial proteins, as ADP/ATP translocase 1 (ANT1) acetylation was not modified by disease (Figure 6C). The 2-fold increase in GDH acetylation in T2D was significantly decreased by honokiol treatment, returning GDH acetylation in T2D back to control levels (Figure 6A). This effect was not present for HADHA, in agreement with the unaltered FA-dependent respiration (Figure 6B).

### Mitochondrial deacetylation increases myocardial energetics and ATP synthesis flux in the T2D heart.

**Figure 7:**
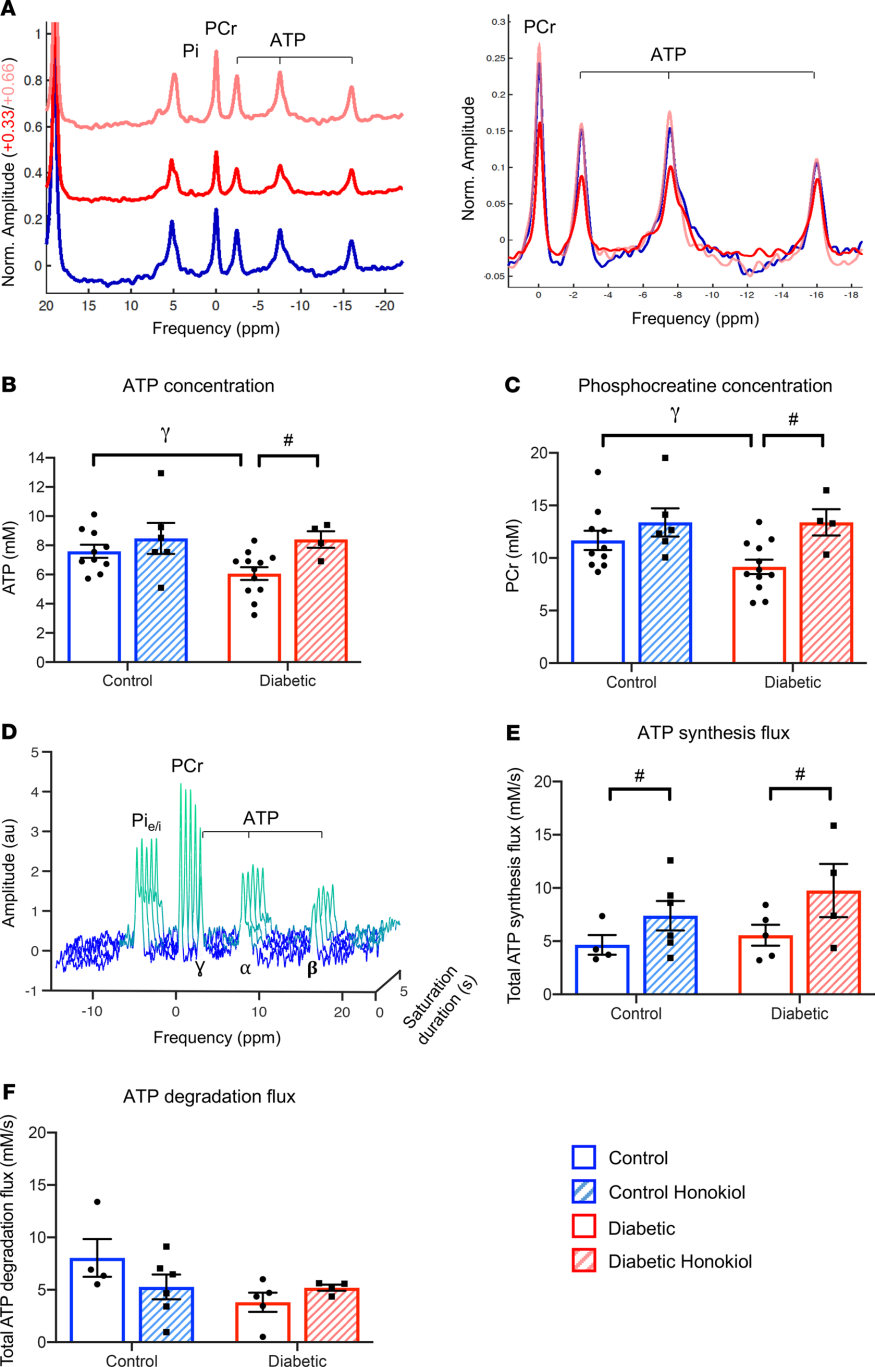
Deacetylation with honokiol restores myocardial energetics and ATP turnover rates in T2D. Myocardial energetics were measured using ^31^P-spectroscopy in the intact contracting heart. Representative spectra are shown for control (blue), diabetic (red), and diabetic rats treated with honokiol (pink) (**A**). Myocardial ATP and PCr concentrations from control and T2D hearts, with or without in vivo honokiol treatment (**B** and **C**). Rates of ATP synthesis and degradation were assessed in the intact heart using saturation transfer. Representative spectra are shown for one of the pair of saturation protocols, in which the γ-P ATP peak is transiently saturated, resulting in the gradual decline in the magnetization of PCr and intracellular Pi (Pi_i_) (**D**). Total ATP synthesis flux and total ATP degradation flux in control and T2D hearts, with and without honokiol treatment (**E** and **F**). ^#^*P* < 0.05 vs. untreated, γ*P* = 0.08 vs. control, analyzed using a 2-way ANOVA with Holm-Sidak’s post hoc correction. T2D, type 2 diabetes; PCr, phosphocreatine.

Given the modeled link between mitochondrial respiratory rates and cardiac energetics, we hypothesized that normalization of mitochondrial protein hyperacetylation in T2D may correct energetic dysfunction at the global cardiac level. ATP and PCr concentrations were, therefore, measured using ^31^P-MRS in these hearts after honokiol treatment. Concentrations of ATP and PCr in contracting T2D hearts were increased by 39% and 46%, respectively, after honokiol treatment (Figure 7, A–C). Double saturation transfer techniques allowed us to measure flux through ATP synthesis and degradation pathways in the intact contracting heart (Figure 7D). There were no significant differences between total ATP synthesis flux and total ATP degradation flux (6.8 ± 0.8 mM/s vs. 5.5 ± 0.6 mM/s, respectively) when all data were summed. Neither diabetes nor honokiol had a significant effect on the forward and reverse rate constants for ATP synthesis or degradation, or on cardiac function (Supplemental Tables 2 and 3). However, honokiol significantly increased total ATP synthesis flux in control and diabetic hearts (*P* < 0.05 effect of honokiol by 2-way ANOVA). In contrast, honokiol had no effect on total ATP degradation flux (Figure 7, E and F). Thus, targeting hyperacetylation rescued myocardial energetics back to control levels and increased total ATP synthesis flux in the T2D heart.

## Discussion

In an early-stage model of T2D, we have demonstrated proportional decreases in both PCr and ATP concentrations, resulting in a normal PCr/ATP ratio. Myocardial energetic dysfunction was accompanied by decreased real-time mitochondrial ATP synthesis rates and decreased mitochondrial respiration rates when oxidizing carbohydrate/amino acid substrates. In T2D, mitochondrial proteins were hyperacetylated, which negatively correlated with state 3 respiration rates. In vivo treatment with honokiol normalized acetylation and restored mitochondrial respiration, associated with increased GDH activity. Honokiol treatment rescued myocardial energetics and increased ATP synthesis flux in T2D. Thus, targeting acetylation provides a mechanism to restore myocardial and mitochondrial energetics in T2D.

### Energetic dysfunction in T2D manifests as decreased high-energy phosphates and localizes to mitochondrial dysfunction.

T2D resulted in proportional decreases in the concentrations of both ATP and PCr, which resulted in a preserved PCr/ATP ratio. This is in agreement with an elegant study by Beer et al., who report decreased concentrations of ATP and PCr of similar magnitude in patients with dilated cardiomyopathy, resulting in no change in the PCr/ATP ratio (22). In both studies, reporting PCr/ATP alone would have overlooked the changes in energy metabolite concentrations and incorrectly indicated that myocardial energetics were preserved, underestimating the true extent of the energetic imbalance within these disease states.

Studies in patients with T2D report decreased PCr/ATP ratios (1); however, our data demonstrated that even before this decrease in PCr/ATP ratio, the T2D heart was already energetically abnormal, and that mitochondria were contributing substantially to this decreased ATP synthesis. Mitochondrial ATP synthesis rates provided real-time flux data through energy generation pathways, and demonstrated that in T2D mitochondrial capacity to synthesize ATP was deranged. As well as identifying whole-heart energetic dysfunction, this study provided the first work to directly associate cardiac energetic dysfunction with reduced rates of mitochondrial ATP synthesis. In addition, these decreases in energy metabolite concentrations were occurring despite normal developed pressure, indicating that energetic dysfunction precedes systolic dysfunction in T2D.

### Acetylation drives mitochondrial respiratory dysfunction in T2D.

The role of mitochondrial protein hyperacetylation within cardiovascular disease is still emerging, but arguably there is no setting more conducive to mitochondrial protein hyperacetylation than the abnormal metabolic milieu of the T2D heart. Data presented here show a 50% increase in mitochondrial acetylation in the T2D heart, with no change to SIRT3 protein levels, indicating that the increased availability of acetyl-CoA and decreased availability of NAD^+^ may be contributory factors (11). Furthermore, in vitro hyperacetylated mitochondria confirm findings that hyperacetylation represses mitochondrial respiration, as when control mitochondria are hyperacetylated to the level found in diabetic mitochondria, the mitochondrial respiratory dysfunction in T2D could be recapitulated.

Of note, when respiring with FAs, no decreases in mitochondrial respiration or mitochondrial ATP synthesis rates were identified in either the SSM or IFM populations under active phosphorylating conditions. This demonstrates that at this early stage of disease, impaired flux through the electron transport chain, Krebs cycle, or β-oxidation pathway were not evident. This is in contrast to more overt/advanced models of T2D, where decreased respiration and ATP/O ratios on FAs are reported (23, 24). This would indicate that as the disease progresses, additional modifications or defects, above and beyond hyperacetylation, occur within the mitochondria.

Decreased respiration and ATP synthesis with carbohydrate/amino acid substrates was in agreement with both patient and animal studies that show downregulation of metabolism of non-FA substrates in T2D (25, 26). Of interest, our mitochondrial energetic defects were only evident when respiring on carbohydrates, whereas the myocardial energetics were impaired when both carbohydrates and FAs were supplied to the heart. This demonstrated that at the higher workload of the intact contracting heart, contributions from both FAs and non-FA sources were required. The healthy heart gains 60% of its ATP from the metabolism of FAs, with the remaining 40% predominantly from carbohydrate and amino acid sources, and in diabetes this shifts further toward FA use. However, the diabetic heart still requires some contribution from these non-FA substrates, and in vivo this would be particularly evident in the fed state, during increased workload, and during ischemia. Thus, in the diabetic heart, under physiological working conditions, both substrates are needed for optimal energetics.

### Honokiol restores acetylation and rescues energetic dysfunction in T2D.

The administration of honokiol for 10 days reversed protein hyperacetylation associated with T2D. This normalization of mitochondrial protein acetylation restored state 3 respiratory rates in T2D mitochondria to control levels, representing an increase of 17%–28%. Strikingly, this is on a similar order of magnitude to improvements obtained in patients with T2D after a 12-week aerobic exercise intervention (27). The improvement in mitochondrial respiration with honokiol results in increases in both PCr and ATP concentrations within the diabetic heart, reverting to concentrations found in control hearts. The saturation transfer experiments allow quantification of flux through these ATP-generating and -consuming pathways, an additional level of insight into myocardial energetics above and beyond that obtained solely from the steady-state concentrations of energy metabolites. Changes in ATP and PCr concentrations are accompanied by increased flux through ATP synthesis pathways in the intact contracting heart, and are independent of changes in ATP degradation pathways, confirming that the beneficial effects of honokiol are via improvements in energy generation.

Previous studies successfully used honokiol in the setting of doxorubicin-induced cardiomyopathy and transverse aortic constriction, demonstrating reduced fibrosis and hypertrophy (18, 19). To date its potential as a therapeutic agent in T2D, a disease characterized by mitochondrial hyperacetylation, to directly improve mitochondrial and myocardial energetics had not been investigated. Pillai et al. report that honokiol could both increase Sirt3 activity and its expression (18); however, in light of our current findings of unchanged SIRT3 protein levels, the latter mechanism appears less influential. This improved respiratory capacity was associated with an increase in GDH activity, as demonstrated by respiratory flux measurements, enzyme assays, and immunoprecipitation of acetylated targets. The increased GDH activity by honokiol treatment occurred via deacetylation of the enzyme, independent of any change in GDH protein levels. That honokiol and deacetylation would be having its greatest effect on GDH was unexpected, as we had initially predicted PDH would be a target for honokiol treatment, but both in vivo and ex vivo measurements of PDH demonstrated this was not the case. In support of our findings, studies from SIRT3 knockout mice demonstrate that hyperacetylation selectively suppresses glutamate respiration (not FA respiration), and one of the greatest fold increases in acetylation is for GDH, which is approximately 90-fold more acetylated than in control hearts (8). Given that GDH is one of the first identified targets of SIRT3 and is acetylated to a far greater extent that other SIRT3 targets (7, 8), this may explain why honokiol exerts its beneficial effect via this mitochondrial target. Of note though, GDH activity is not suppressed by diabetes alone, indicating that the effects of honokiol to increase GDH activity in diabetes may be providing additional metabolic pathway flux to circumvent another defect (potentially decreased PDH activity) in diabetes.

In conclusion, restoration of mitochondrial respiratory rates through pharmacological deacetylation corrected the myocardial energetic dysfunction identified in the T2D heart. This correction occurred through increases in the concentrations of both ATP and PCr, as well as improvement in ATP synthesis flux in the T2D heart. This provides strong evidence that the energetic dysfunction within the T2D heart at this early stage of the disease is primarily caused by hyperacetylation-mediated reductions in mitochondrial respiratory rates. This work therefore identifies and corrects a form of mitochondrial dysfunction within the T2D heart and presents an attractive clinical target with the potential to improve cardiovascular mortality within T2D.

## Methods

### Rat model of T2D.

T2D was induced as previously described (11), generating a mild model of the disease presenting with insulin resistance, mild hyperglycemia, hyperinsulinemia, and hyperlipidemia. This model was chosen as it avoids the extreme hyperglycemia present in other models, more closely resembling the majority of patients with T2D, and at this stage of disease has no systolic dysfunction or hypertrophy (28). Briefly, male Wistar rats (Envigo) were fed a high-fat diet ad libitum (Special Diet Services, 829197, 60% calories from fat), and on day 14 they received a single low-dose i.p. injection of streptozotocin (25 mg/kg body weight, w/w in citrate buffer, pH 4). Control rats were fed standard chow diet, and rats were maintained on their respective diets for a further 5 weeks. Rats were terminally anesthetized using an i.p. injection of pentobarbital sodium (0.7 mL of 200 mg/mL Euthatal).

### Honokiol administration.

Honokiol was first dissolved in DMSO at 36 mg/mL and then resuspended in corn oil to achieve a final concentration of 2 mg/mL. The weights of the rats were taken daily, and honokiol or vehicle (5.6% DMSO in corn oil) was administered at a dose of 0.4 mg/kg via i.p. injection at 4 pm daily for the final 10 days of the experimental protocol (adapted from ref. 18).

### ^31^P-MRS for cardiac energetics.

Rats were terminally anesthetized, and the hearts excised and arrested in ice-cold Krebs-Henseleit (KH) buffer. The aorta was dissected free and then cannulated before retrograde Langendorff perfusion with KH buffer supplemented with glucose (11 mM), albumin (1.5%), and palmitate (0.4 mM). Hearts were perfused at a constant hydrostatic pressure of 100 mmHg, an end-diastolic pressure of 3–5 mmHg, oxygenated with 95% O_2_, 5% CO_2_, and maintained at 37°C throughout. A balloon containing phenylphosphonic acid (PPA, 10 mM) was inserted into the left ventricle to allow absolute quantification of energy metabolites. The contracting heart was placed into a glass NMR sample tube and lowered into the isocentre of an 11.7 T vertical bore preclinical MRI scanner (Magnex Scientific) with temperature control supplemented by forced air at 37°C. Fully relaxed ^31^P spectra were acquired through a simple pulse/acquire acquisition sequence (10-second TR, 32 averages, 90° flip angle, 10 kHz bandwidth, 1024 points). Following this protocol, 3 further pulse/acquire measurements (10-second TR, 16 averages, 90° flip angle, 10 kHz bandwidth, 1024 points) were carried out to generate a standard curve for phosphorus with the internal balloon remotely filled with an additional 100 μl of 10 mM PPA after each scan. Acquired MR spectra were quantified using open-source spectral analysis software (29, 30), and absolute quantification was performed taking into account heart mass and extracellular volume fraction.

Separately, the forward and reverse fluxes of ATP synthesis and degradation were measured through saturation transfer experiments (31). Specifically, the rate constants *k_f_*, *k’_f_* and (*k_r_+k’_r_*) can be determined in the following reaction via 2 separate MR experiments:

 (Equation 1).
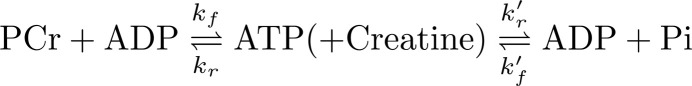


In the first experiment, the saturation of γ-ATP was varied for lengths of time, followed by the observation of the resulting decrease in PCr and intracellular inorganic P (Pi) signal produced via label exchange, and, in the second experiment, PCr and Pi were simultaneously saturated, with the subsequent observation of the decrease in γ-ATP. By fitting these curves to an appropriate mathematical model that is obtained from the integrated solutions to the Bloch equations, the rate constants *k_f_*, *k’_f_* can be determined from the first experiment and (*k_r_+k’_r_*) from the second experiment. This then permits the determination of the forward and reverse rates of ATP synthesis and ATP degradation; i.e., [PCr]*k_f_* + [Pi]*k’_f_* and [ATP] (*k_r_+k’_r_*). Here, we performed both saturation transfer experiments using either (a) a 27-ms-long modified SNEEZE pulse using a DANTE-like chain of 183, 90, 23, 11, 6, and 0 pulses corresponding to a saturation duration of 4.94, 2.43, 0.62, 0.30, 0.13, and 0 seconds or (b) a dual-band quasi-adiabatic saturation pulse designed via a hybrid optimal control/SLR algorithm approach that was 25 milliseconds in duration and iterated the same number of times as before, providing complete saturation of PCr and Pi for a duration of 4.58, 2.25, 0.58, 0.28, 0.15, and 0 seconds. In both cases, a hard 90^o^ pulse excitation was used, with 16 averages, 1024 complex points, and a 10 kHz bandwidth. Further details are provided in Supplemental Methods.

### Mitochondrial isolation and respiration.

SSM and IFM were isolated from the heart as described previously (32) and in the presence of deacetylation inhibitors (10 mM nicotinamide and 500 nM Trichostatin A, MilliporeSigma). Mitochondria were respired as previously described (32), with either a carbohydrate/amino acid mix containing glutamate (20 mM), pyruvate (10 mM), and malate (5 mM) or a FA mix containing GPM with palmitoyl-carnitine (10 μM) as cosubstrate. State 3 respiration was induced using ADP (MilliporeSigma) (200 μM).

### ^31^P-MRS for mitochondrial ATP synthesis rates.

Isolated mitochondria were suspended in respiratory media with substrates as per the respiration experiments, and maintained at 37°C in an NMR tube placed in the 11.7 T magnet. ^31^P spectra were acquired through a simple pulse/acquire acquisition sequence (0.25-second TR, 60 averages, 25^o^ flip angle hard pulse excitation, 10 kHz acquisition, 1024 points), giving one spectra every 15 seconds. Two minutes after the start of the acquisition, ADP (5 mM) was injected via a drugs line into the magnet, and synthesis of ATP by the isolated mitochondria was monitored in real time at 37^o^C for a further 20 minutes. Spectra were analyzed using the AMARES algorithm in the jMRUI software package, and as shown in Figure 2, the β-ATP peak (as this was unique to ATP) was used for the quantification of the maximal ATP synthesis rates by least-squares nonlinear fitting of concentrations to a modified Hill Function (further details are provided in Supplemental Methods).

### Hyperpolarized ^13^C-MRS to assess in vivo PDH flux.

Hyperpolarized experiments were performed on a 7 T MRI system (Varian) as previously described (28). Briefly, rats were anesthetized with 2% isoflurane in oxygen and 1 mL of 80 mM hyperpolarized [1-^13^C] pyruvate was injected into the tail vein over 10 seconds. ^13^C-MR spectra were acquired from the heart (10-mm axial slab) gated to the R-wave of the ECG, approximately every second for 60 seconds, using a 72-mm dual-tuned birdcage volume transmit ^1^H/^13^C coil and a ^13^C 2-channel surface receive coil (Rapid Biomedical; 15° hard pulse, 13.0 kHz bandwidth). PDH flux was determined by the metabolism of [1-^13^C] pyruvate into [1-^13^C] bicarbonate within the myocardium, expressed as bicarbonate/pyruvate ratio from the sum of 30 seconds of data starting from the first appearance of the pyruvate peak.

### In vitro mitochondrial acetylation.

A stock solution of acetic anhydride (2 mM) was prepared in acetonitrile (1 M). SSM and IFM (1.6 mg of mitochondrial protein) were added to 7 μM, 12 μM, and 17 μM acetic anhydride. After inversion for 6 minutes, lysine (9 mM) was added to fully quench the reaction. The resulting sample was centrifuged at 3020*g* for 5 minutes at 4°C, and the pellet resuspended in mitochondrial isolation solution before respiration measurements (13, 33).

### In vitro biochemical assays.

Freeze-thawed mitochondria (10 μg protein) were assayed for PDH and MDH activities according to manufacturer’s protocols (MilliporeSigma). GDH activity was measured using a protocol adapted from Passonneau and Lowry (34), using α-ketoglutarate (2 mM), ammonium acetate (25 mM), ADP (100 μM), and NADH (200 μM).

### Western blotting.

Mitochondrial lysed protein (30 μg) was loaded onto an SDS-PAGE gel (NuPAGE 4%–12% Bis-Tris Midi Protein Gels, 20 well, Invitrogen) and separated by electrophoresis and then transferred onto nitrocellulose membranes. Membranes were incubated overnight at 4°C with primary antibody, and bands were quantified using LI-COR C-Digit technology. Pan-acetyl-lysine antibody was purchased from Cell Signaling Technology (9441S, 9681S), the GDH antibody (Ab166618) and secondary antibodies (Ab6721, Ab97023) were purchased from Abcam.

### Coimmunoprecipitation protocol.

Mitochondrial (SSM) protein lysates were incubated in CHAPS buffer (KCl [120 mM], HEPES [20 mM], MgCl_2_ [5 mM], EGTA [1 mM], pH 7.2, supplemented with fat-free BSA [5 mg/mL]) overnight at 4°C with rabbit acetyl-lysine antibody. Immunocaptured proteins were then isolated using Protein-G agarose beads (Cell Signaling Technology), washed multiple times with CHAPS buffer, and then eluted in sample buffer at 95°C. Samples were then separated on 4%–12% Bis-Tris gels, and probed with the appropriate antibodies. Protein expression was analyzed using the following primary antibodies: rabbit acetyl-lysine, rabbit GDH (12793 Cell Signaling), rabbit ADP/ATP translocase (ANT1, custom made by Eurogentec), and rabbit hydroxyacyl-CoA dehydrogenase (HADHA) from Protein-Tech (10758-1-AP). Protein densitometry was measured using NIH ImageJ software.

### Statistics.

Results are presented as mean ± SEM and considered significant at *P* values of less than 0.05 using GraphPad Prism 8.0.0. Data sets containing 2 groups were analyzed using the 2-tailed parametric unpaired *t* test. Data sets containing multiple groups (control vs. diabetic) and multiple variables (vehicle vs. honokiol) were analyzed using a 2-way ANOVA, with Holm-Sidak’s post hoc correction for multiple comparisons. Data sets with 3 groups were analyzed using a 1-way ANOVA, with Tukey’s post hoc correction for multiple comparisons.

### Study approval.

All animal experiments conformed to the UK Home Office Guidance on the Operation of the Animals (Scientific Procedures) Act, 1986, and were approved by the University of Oxford ethics committee.

## Author contributions

MK, SS, DT, CNMA, and IS researched the data. JJM and KNT analyzed the data, DJT and LCH researched and analyzed the data. MK and LCH wrote the manuscript. All authors reviewed the manuscript.

## Supplementary Material

Supplemental data

## Version 1 09/03/2020

Electronic publication
