# Supplementary material for: Rescue of myocardial energetic dysfunction in diabetes through the correction of mitochondrial hyperacetylation by honokiol
Source: JCI Insight. 2020 Sep 3;5(17):e140326. doi: 10.1172/jci.insight.140326 (PMC7526448; doi:10.1172/jci.insight.140326)
Supplement: Supplemental data [file jciinsight-5-140326-s226.pdf]

Supplementary Table 1. Physical characteristics and blood glucose concentrations from control and diabetic rats, with and without honokiol treatment.

	Control	Control Honokiol	Diabetic	Diabetic Honokiol
Fed blood glucose (mM)	8.37 ± 0.35	7.53 ± 0.32	9.99 ± 0.37 *	9.81 ± 0.47 *
Fasting blood glucose (mM)	6.06 ± 0.18	6.30 ± 0.37	7.86 ± 0.37 *	7.48 ± 0.36 *
Epididymal fat pad weight (g)	7.3 ± 0.8	8.4 ± 1.2	10.7 ± 1.1 †	11.7 ± 1.3 †
Body weight (g)	379 ± 16	377 ± 10	411 ± 10 †	414 ± 16 †
Heart weight to body weight ratio (mg/g)	2.28 ± 0.05	2.28 ± 0.12	2.45 ± 0.06	2.34 ± 0.04

Epididymal fat pad weight is used as an indicator of adiposity. n = 9 per group. * $p < 0.05$ vs. respective control group. † $p < 0.05$ vs control group without multiple comparisons.

Supplementary Table 2. Rate constants for ATP synthesis and degradation in control and diabetic hearts, with and without honokiol treatment.

	Control (n = 4)	Control Honokiol (n = 6)	Diabetic (n = 5)	Diabetic Honokiol (n = 4)
k_f	0.30 ± 0.04	0.37 ± 0.06	0.23 ± 0.04	0.24 ± 0.04
k'_f	0.21 ± 0.08	0.32 ± 0.08	0.39 ± 0.11	0.43 ± 0.13
$k_r + k'_r$	1.07 ± 0.25	0.55 ± 0.11	0.59 ± 0.10	0.62 ± 0.05

Supplementary Table 3. Cardiac function in control and diabetic rats, with and without honokiol treatment.

	Control (n = 11)	Control Honokiol (n = 5)	Diabetic (n = 11)	Diabetic Honokiol (n = 4)
Heart Rate (bpm)	227 ± 17	192 ± 19	200 ± 13	193 ± 25
Developed pressure (mmHg)	112 ± 8	126 ± 13	114 ± 12	148 ± 18
Rate Pressure Product (mmHg/min x 10^3)	25.4 ± 0.2	24.9 ± 0.2	22.5 ± 0.2	27.6 ± 0.3

Supplementary Figure 1

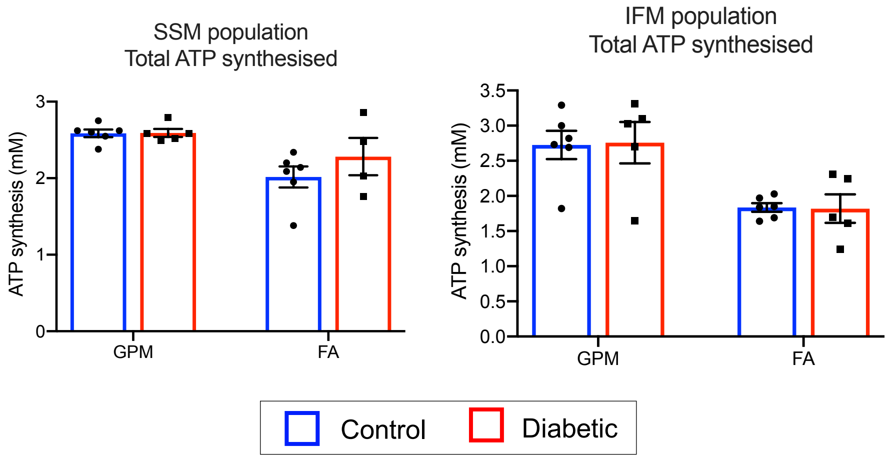

Supplementary Figure 1. Total ATP synthesised over the course of the experiment by subsarcolemmal (SSM) and interfibrillar (IFM) mitochondria from control and T2D hearts. Mitochondrial ATP synthesis was measured by respiring mitochondria in an 11.7 T magnet and using ^{31}P- spectroscopy. Mitochondria are respired with carbohydrates and amino acid (Glutamate, Pyruvate, Malate – GPM) or fatty acids (FA).

Supplementary Figure 2

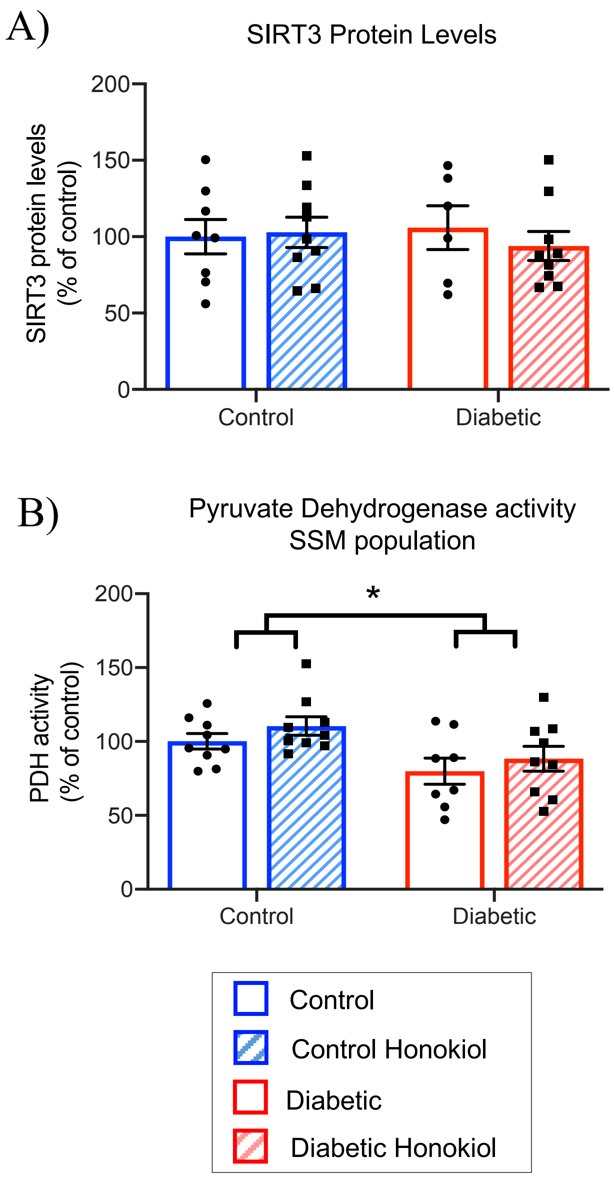

Supplementary Figure 2. SIRT3 protein levels from control and T2D hearts, following *in vivo* honokiol or vehicle treatment (A). Pyruvate dehydrogenase activity in cardiac subsarcolemmal (SSM) mitochondria from control and T2D rats, following *in vivo* honokiol or vehicle treatment (B). * $p < 0.05$ diabetic groups vs. control groups, by two-way ANOVA.

Supplementary Figure 3

Acetylation representative images

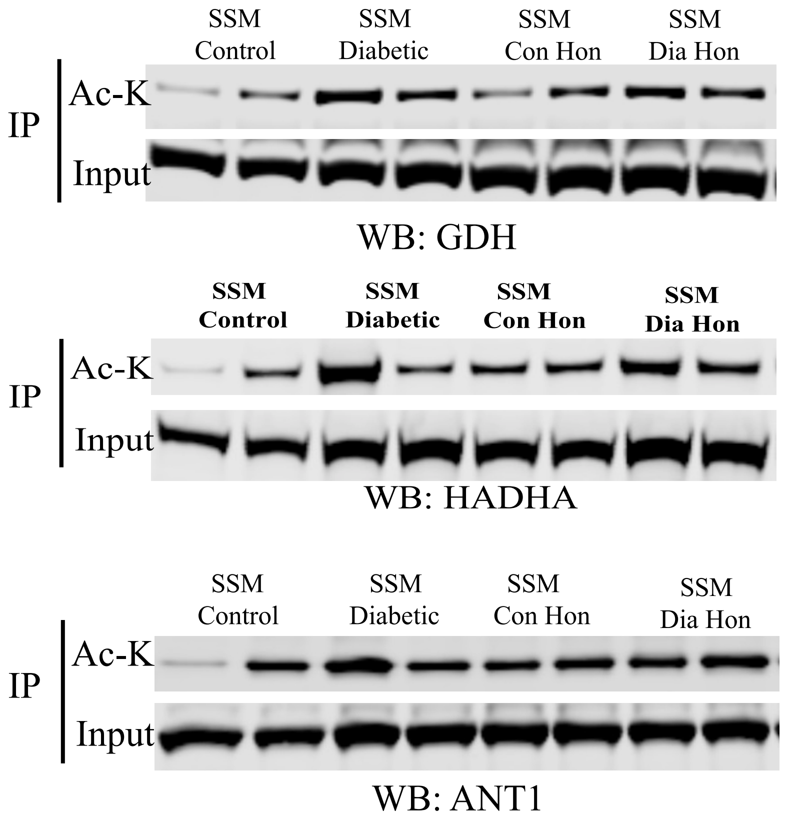

Supp Figure 3. Representative images from western blotting for acetylation of mitochondrial proteins. GDH; Glutamate dehydrogenase, HADHA; hydroxyacyl-CoA dehydrogenase, ANT1: ADP/ATP translocase 1.

Supplementary methods

Further details for ^{31}P-Magnetic Resonance Spectroscopy (^{31}P-MRS) for cardiac energetics, for saturation transfer experiment analysis

Here, we performed both saturation-transfer experiments using either (1) a 27 ms-long modified SNEEZE pulse using a DANTE-like chain of [183, 90, 23, 11, 6, 0] pulses corresponding to a saturation duration of [4.94, 2.43, 0.62, 0.30, 0.13, 0] seconds; or (2) a dual-band quasi-adiabatic saturation pulse designed via a hybrid optimal control / SLR algorithm approach that was 25 ms in duration, and iterated the same number of times as before, providing complete saturation of PCr and Pi for a duration of [4.58, 2.25, 0.58, 0.28, 0.15, 0] s. In both cases, a hard 90º pulse excitation was used, with 16 averages, 1024 complex points, and a 10 kHz bandwidth. Spectral quality was assessed via the Cramér-Rao Lower Bound returned on spectral quantification, and all spectra were included in subsequent analysis. The nonlinear fitting of returned amplitudes was performed in a least-squares sense to the expression

$$y(t) = M_0(1 - (k\,\tau(1 - e^{-t/\tau})))$$

via a constrained global optimisation algorithm that explicitly included the spin-physics constraint that

$$\tau - k \geq 0$$

where *k* is the rate constant of interest, and

$$\tau = \frac{1}{T_1} + k$$

Further details for ^{31}P-Magnetic Resonance Spectroscopy (^{31}P-MRS) for mitochondrial energetics analysis

Spectra were analysed using the AMARES algorithm, and as shown in Figure 2, the β-ATP peak was used for quantification of ATP synthesis rates by the least-squares nonlinear fitting of concentrations to a modified Hill Function of the form

$$H(t) = a + \frac{b-a}{1 + \left(\frac{t}{c}\right)^{-n}}$$

This permits the determination of the maximum rate of ATP synthesis, as it can analytically be shown that the maximum rate of product production occurs at a time given by

$$\Re\left(c e^{-\frac{i\pi}{n}} (1-n)^{\frac{1}{n}} (n+1)^{-1/n}\right)$$

with the corresponding value determined from the fit.
